# Supplementary material for: Conditional chemoconnectomics (cCCTomics) as a strategy for efficient and conditional targeting of chemical transmission
Source: eLife. 2024 Apr 30;12:RP91927. doi: 10.7554/eLife.91927 (PMC11060718; doi:10.7554/eLife.91927)
Supplement: Figure 3—source data 3. [file elife-91927-fig3-data3.zip › Figure 3 source data3 part 2.zip!/Figure 3-Source data 3-part2/1053f2-1-CRISPResso_Report_y3hlioj1.zip!/CRISPResso_on_1053f2_1/1c.Alignment_barplot.pdf]

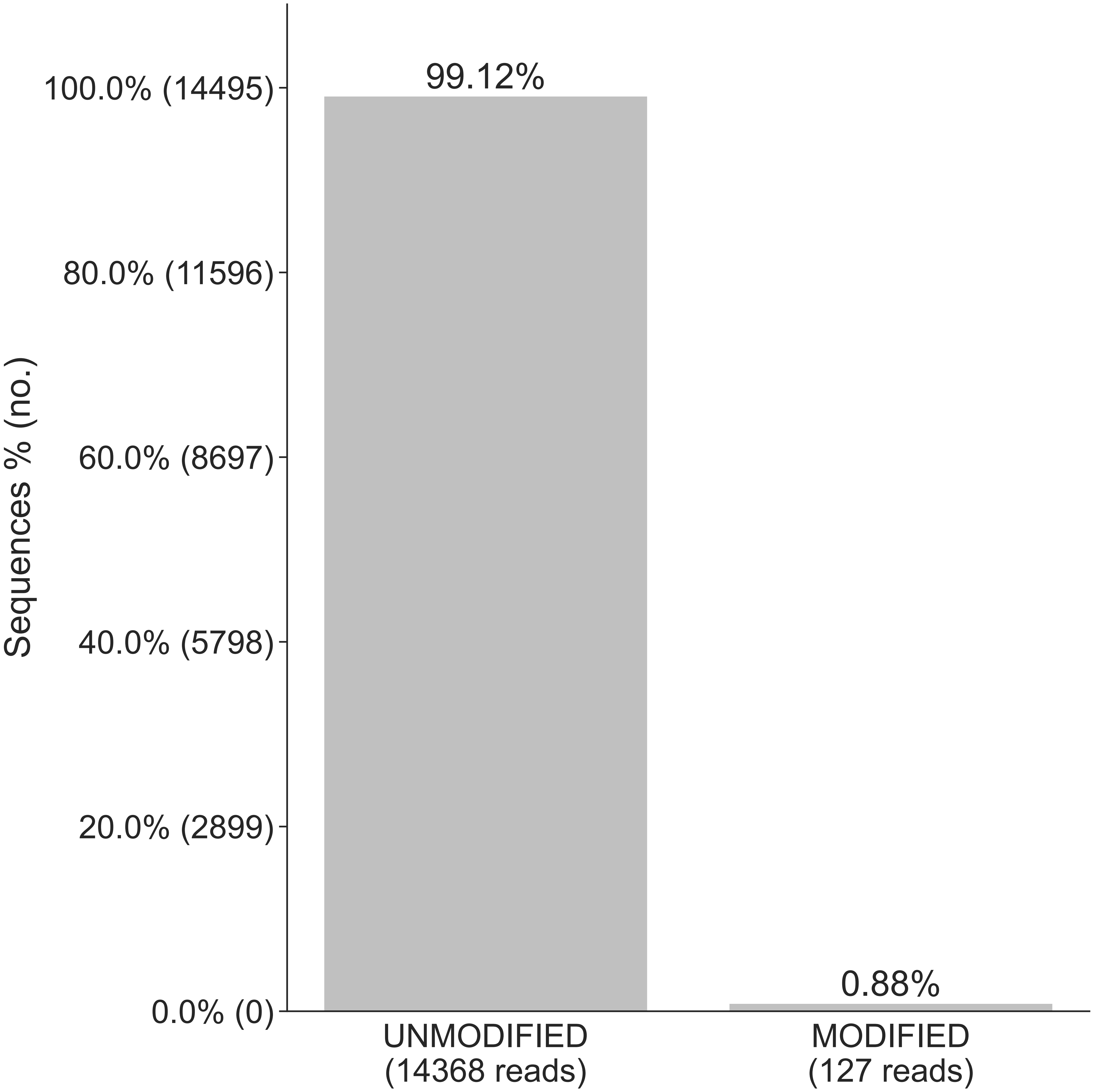

Sequences % (no.)
100.0% (14495)
80.0% (11596)
60.0% (8697)
40.0% (5798)
20.0% (2899)
0.0% (0)
99.12%
0.88%
UNMODIFIED
(14368 reads)
MODIFIED
(127 reads)
